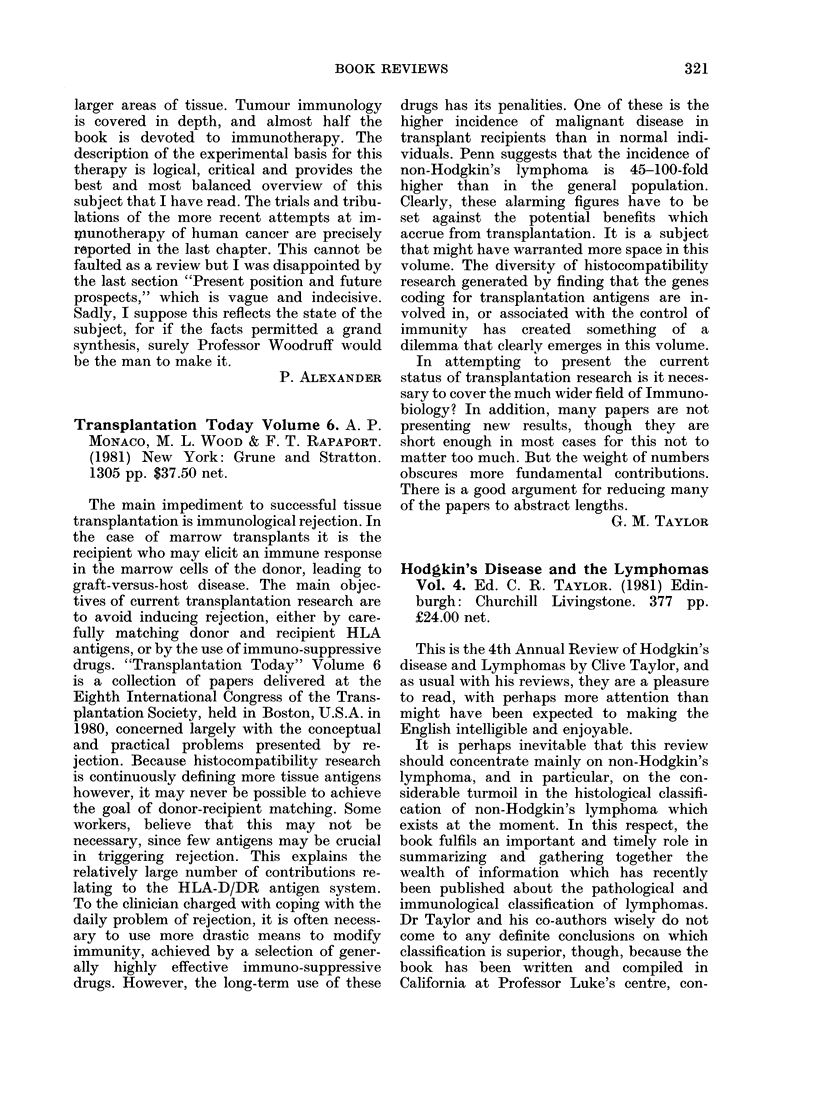# Transplantation Today Volume 6

**Published:** 1982-02

**Authors:** G. M. Taylor


**Transplantation Today Volume 6.** A. P. MONACO, M. L. WOOD & F. T. RAPAPORT. (1981) New York: Grune and Stratton. 1305 pp. $37.50 net.

The main impediment to successful tissue transplantation is immunological rejection. In the case of marrow transplants it is the recipient who may elicit an immune response in the marrow cells of the donor, leading to graft-versus-host disease. The main objectives of current transplantation research are to avoid inducing rejection, either by carefully matching donor and recipient HLA antigens, or by the use of immuno-suppressive drugs. "Transplantation Today" Volume 6 is a collection of papers delivered at the Eighth International Congress of the Transplantation Society, held in Boston, U.S.A. in 1980, concerned largely with the conceptual and practical problems presented by rejection. Because histocompatibility research is continuously defining more tissue antigens however, it may never be possible to achieve the goal of donor-recipient matching. Some workers, believe that this may not be necessary, since few antigens may be crucial in triggering rejection. This explains the relatively large number of contributions relating to the HLA-D/DR antigen system. To the clinician charged with coping with the daily problem of rejection, it is often necessary to use more drastic means to modify immunity, achieved by a selection of generally highly effective immuno-suppressive drugs. However, the long-term use of these drugs has its penalities. One of these is the higher incidence of malignant disease in transplant recipients than in normal individuals. Penn suggests that the incidence of non-Hodgkin's lymphoma is 45–100-fold higher than in the general population. Clearly, these alarming figures have to be set against the potential benefits which accrue from transplantation. It is a subject that might have warranted more space in this volume. The diversity of histocompatibility research generated by finding that the genes coding for transplantation antigens are involved in, or associated with the control of immunity has created something of a dilemma that clearly emerges in this volume.

In attempting to present the current status of transplantation research is it necessary to cover the much wider field of Immunobiology? In addition, many papers are not presenting new results, though they are short enough in most cases for this not to matter too much. But the weight of numbers obscures more fundamental contributions. There is a good argument for reducing many of the papers to abstract lengths.

G. M. TAYLOR